# Uncovering the breeding contribution of transposable elements from landraces to improved varieties through pan-genome-wide analysis in rice

**DOI:** 10.3389/fpls.2025.1573546

**Published:** 2025-04-14

**Authors:** Xiaoxia Li, Xiaofan Dai, Huiying He, Wu Chen, Qian Qian, Lianguang Shang, Longbiao Guo, Wenchuang He

**Affiliations:** ^1^ Rice Research Institute, Shenyang Agricultural University, Shenyang, China; ^2^ Shenzhen Branch, Guangdong Laboratory of Lingnan Modern Agriculture, Genome Analysis Laboratory of the Ministry of Agriculture and Rural Affairs, Agricultural Genomics Institute at Shenzhen, Chinese Academy of Agricultural Sciences, Shenzhen, China; ^3^ Yazhouwan National Laboratory, Sanya, Hainan, China; ^4^ State Key Laboratory of Rice Biology and Breeding, China National Rice Research Institute, Hangzhou, China

**Keywords:** transposable element, super pan-genome, improvement, rice, molecular breeding

## Abstract

**Introduction:**

The rice improvement process, driven by modern breeding techniques, represents the second revolutionary advancement in rice agronomic traits, following domestication. Advances in pan-genomes and enhanced capacity for analyzing structural variations have increasingly highlighted their role in rice genetic improvement. Transposable element (TE) variants have been previously reported to influence rice genomic diversity during the domestication, but their contribution to the improvement from landraces to improved varieties remains unclear.

**Methods:**

Here, we combined a high-quality pan-TE variation map, transcriptome profiles, and phenotypic data for 100 landraces and 92 improved varieties to investigate the contribution of TE variations to phenotypic improvement in rice.

**Results:**

The total number and length of TE variations in improved varieties were significantly greater than those in rice landraces, particularly for Ty3-retrotransposons, LTR *Copia* and *Helitron* elements. Comparing landraces and improved varieties, 4,334 selective TEs were detected within or near 3,070 genes that were enriched in basic metabolism and development and stress resistance. Among the 14,076 differentially expressed genes between the two groups, the expression level of 3,480 (24.7%) genes were significantly associated with TE variations. Combining with haplotype analysis, we demonstrated potential patterns of how TEs affect gene expression variation and thereby participate in the improvement of important agronomic traits in rice.

**Discussion:**

Collectively, our results highlight the contributions of TE variations to rice improvement in shaping the genetic basis of modern rice varieties and will facilitate the exploration of superior genes and advance molecular breeding efforts in rice.

## Introduction

Rice serves as a staple food crop for nearly half of the global population (Wing et al., 2018), and enhancing rice productivity is essential for meeting the growing demands of the ever-increasing world population (Shang et al., 2022; Tao et al., 2019). Approximately 10,000 years ago, the domestication of rice commenced (Huang and Han, 2015). This process significantly shaped the initial morphology and panicle traits of cultivated rice, such as Asian cultivated rice (*Oryza sativa*, *Os*) encompassing two main subspecies: *Oryza sativa* ssp*. japonic*a (*Osj*) and *Oryza sativa* ssp. *indica* (*Osi*), transforming it from its wild progenitors (Wang et al., 2018). Key modifications included reduced seed shattering, increased grain size, modified plant architecture, altered awn length, and enhanced seed dormancy, thereby rendering it more suitable for human cultivation practices. For instance, a mutation in the *sh4* gene resulted in reduced or eliminated seed shattering during the domestication, enabling seeds to remain attached to the panicle after maturity, thereby facilitating human harvesting (Li et al., 2006).

Rice landraces, serving as domesticated derivatives from the wild progenitors, demonstrate significant abiotic stress tolerance and biotic resistance. However, these traditional genotypes exhibit notable agronomic constraints including limited yield capacity, predisposition to lodging, and photoperiodic sensitivity that substantially reduce agricultural productivity (Li et al., 2023). To address these limitations, systematic genetic improvement initiatives emerged during the mid-twentieth century, gaining momentum through the 1960s Green Revolution (Stone and Glover, 2017). This improvement process in rice strategically utilized diverse germplasm resources to develop improved varieties through trait-based selection (Li et al., 2023; Hour et al., 2020). Breeding priorities focused on optimizing plant architecture, enhancing yield potential, improving stress resilience, regulating phenological phases, and refining grain quality parameters (Yamasaki and Ideta, 2013). A landmark advancement involved the *sd1* locus mutation that disrupts gibberellin biosynthesis, effectively converting tall, lodging-susceptible landraces into semi-dwarf cultivars with improved yield stability (Sasaki et al., 2002). Prolonged breeding efforts have resulted in substantial genomic differences between landraces and improved varieties and identified a series of beneficial genes such as those related to drought tolerance (Li et al., 2023), photoperiod sensitivity (Hour et al., 2020; Nguyen and Nhan, 2024), and grain protein content (Shi et al., 2023). However, these studies predominantly relied on small genetic variations, such as single nucleotide polymorphisms (SNPs), derived from Illumina short-read sequencing, which are insufficient to capture complex structural variations, including copy number variations, inversion, and other genomic rearrangements.

Transposable elements (TEs) are ubiquitous in plant genomes and serve as major drivers of genomic variation and species diversity (Feschotte et al., 2002). In the mid-twentieth century, Barbara McClintock demonstrated that TEs are associated with color variegation in maize kernels and leaves (McClintock, 1951), highlighting their critical role in phenotypic variation. TEs are classified into two major categories based on their transposition mechanisms: retrotransposons and DNA transposons. Retrotransposons are further divided into those with long terminal repeats (LTRs), such as Ty3-retrotransposons, LTR *Copia* and unknown families, and non-LTR elements, including long interspersed nuclear elements (LINEs) and short interspersed nuclear elements (SINEs). DNA transposons encompass various families, including miniature inverted repeat transposable elements (MITEs, including *Stowaway* and *Tourist*), DTC (*CACTA*), DTA (*hAT*), DTT (*Tc1-Mariner*), DTM (*Mutator*), DTH (*PIF-Harbinger*), and *Helitron* elements (Wicker et al., 2007). For example, a LTR insertion (INS) in the *GY3* promoter suppresses its expression, leading to elevated levels of active cytokinins in young panicles (Wu et al., 2023). Similarly, a *Helitron* INS in the *MYB61* promoter has been shown to influence rice nitrogen utilization and grain yield (Gao et al., 2020). The DTH transposon-derived gene *PANDA* epigenetically regulates panicle number and grain size in rice (Mao et al., 2022). Despite these findings, the impact of TE variations on the improvement of rice varieties—ranging from landraces to improved varieties—remains underexplored.

Recently released rice super pan-genome consisting of 251 wild and cultivated rice genomes allows effectively construction of a high-quality pan-TE map across diverse rice germplasms (Shang et al., 2022; Li et al., 2024). Our previous study has discussed the functional effects of TE variations to rice domestication from the wild rice to cultivated rice accessions (Li et al., 2024). In this study, we analyzed the characteristics and differences in TE variations between rice landraces and improved varieties using a pan-TE map comprising 100 landraces and 92 improved varieties. By integrating transcriptome profiles and phenotypic data, we elucidated the potential mechanisms by which TE variations influence gene expression and agronomic traits during rice improvement. Collectively, our findings underscore the contributions of TE variations to population adaptation, agronomic trait enhancement, gene cloning, and molecular breeding in rice.

## Results

### Differences in TE variation between rice landraces and improved varieties

**Figure 1:**
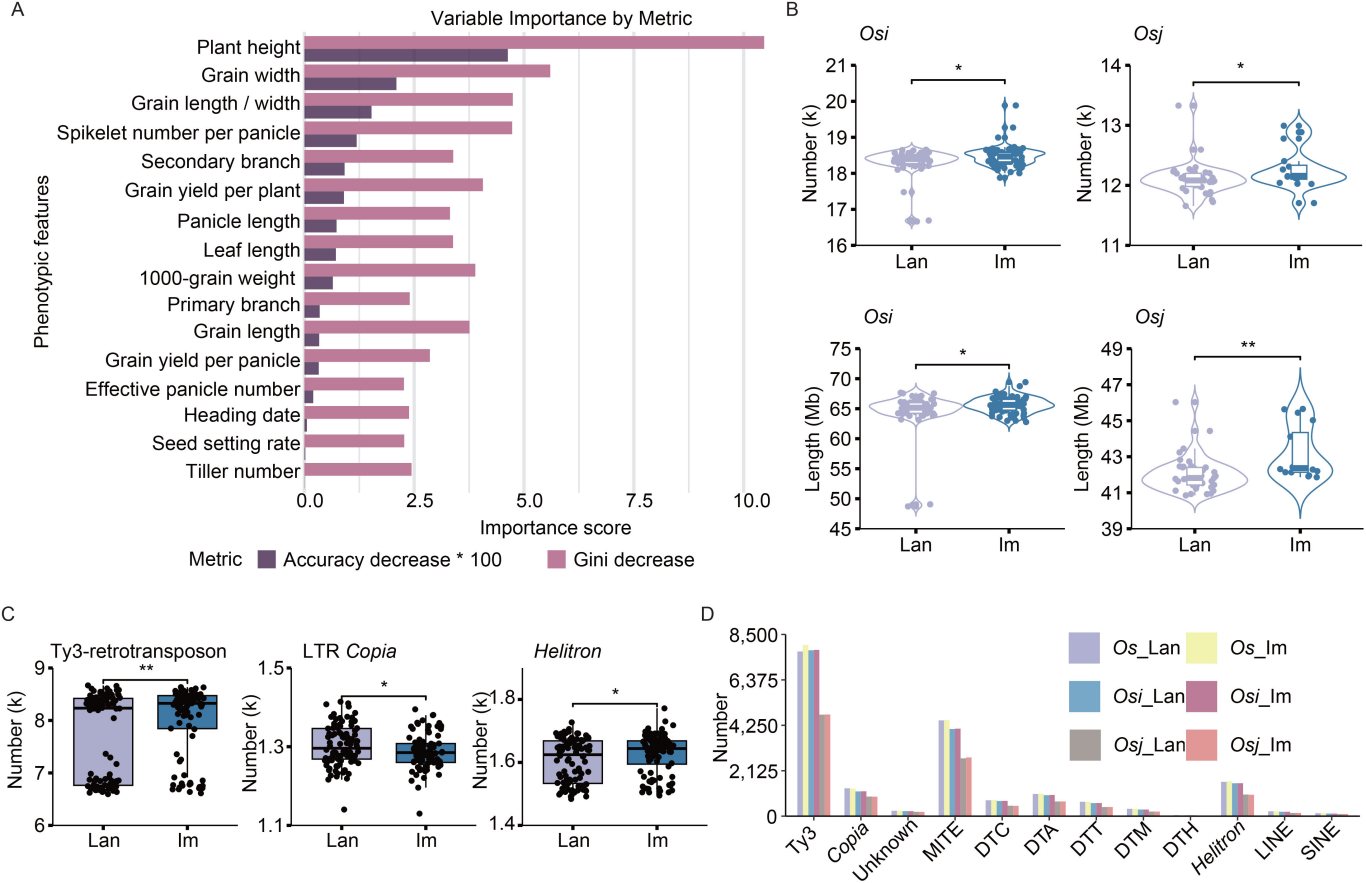
Differences in TE variation between landraces and improved varieties. **(A)** Importance ranking of agronomic traits for rice improvement between landraces and improved varieties, assessed through permutation-based accuracy reduction and Gini impurity decrease. **(B)** Differences in the total number and length of TE variations per accession across different rice subpopulations. Lan and Im denote landrace and improved varieties, respectively. *Osi* and *Osj* refer to *O. sativa indica* and *O. sativa japonica*, respectively. **(C)** Differences in the total number of TE variations for Ty3-retrotransposons, LTR *Copia*, and *Helitron* elements in each landrace and improved variety across all *Os* accessions. **(D)** Total number of TE variations for different TE families across subpopulations. Unknown and refer to LTR/unknown family and Ty3-retrotransposons, respectively. *Os*_lan, *Os*_im, *Osi*_lan, *Osi*_im, *Osj*_lan, and *Osj*_im refer to *Os* landraces; improved varieties of *Os*, *Osi* landraces; improved varieties of *Osi*, *Osj* landraces; and improved varieties of *Osj*, respectively. Significance was determined using Student’s *t*-test, **p < 0.01, *p < 0.05.

We collected 192 *Os* accessions, including 100 landraces (*Os*_lan) and 92 improved varieties (*Os*_im) (**Supplementary Table S1**). To assess phenotypic differences between landraces and improved varieties, we constructed a binary classification prediction model based on quantified phenotypic traits through the Random Forest (RF) algorithm, a robust statistical learning approach for multidimensional feature analysis. The model achieved a high prediction accuracy of 89.5% (95% CI, 75.2%–97.1%) and identified plant height as the trait most significantly affected by rice improvement, followed by grain width, grain length-to-width ratio, spikelet number per panicle, secondary branch number, and grain yield per plant (**Figure 1A**, **Supplementary Figure S1**). Among them, 58 *Osi* landraces (*Osi*_lan), 39 *Osj* landraces (*Osj*_lan), 71 *Osi* improved varieties (*Osi*_im), and 16 *Osj* improved varieties (*Osj*_im) were selected for the subsequent population comparison analysis.

To investigate TE distribution differences, we utilized a high-quality pan-TE map constructed from 221 wild and cultivated rice genomes in our previous study (Li et al., 2024). For the TE variants, genotypes that are consistent with those of the outgroup species (including *O. glaberrima*, *O. barthii*, and *O. glumaepatula*) were designated as ancestral genotypes (**Supplementary Figure S2**). Conversely, genotypes exhibiting alleles different from those of the outgroup species were classified as derived genotypes, referred to as dTEs. A total of 65,346 and 62,678 non-redundant dTEs were finally retained for the 100 landraces and 92 improved varieties, respectively (**Supplementary Figure S3A**). Specifically, 50,446, 55,033, 32,028, and 25,735 dTEs were detected in *Osi*_lan, *Osj*_lan, *Osi*_im, and *Osj*_im, respectively (**Supplementary Figure S3A**). To account for subpopulation size differences, we calculated the characteristics for each accession, revealing that improved varieties exhibited significantly greater dTE numbers and lengths compared to landraces (*p* < 0.05, **Figure 1B**, **Supplementary Figure S3B**), especially the dTE insertion events (**Supplementary Figure S3C**). These findings suggest that TE variations may play a pivotal role in the rice improvement process.

Comparative analysis of different TE families revealed significantly elevated abundances of Ty3-retrotransposons, LTR *Copia*, *Helitron*, and DTC elements in each improved variety compared to landrace variety (**Figure 1C**, **Supplementary Figure S3D**). The dTEs profiles of both germplasm types were predominantly composed of Ty3-retrotransposons, MITEs, and *Helitron* elements (**Figure 1D**), consistent with their substantial representation in the rice genome.

### Contribution of TE variations to divergent selection during rice improvement

**Figure 2:**
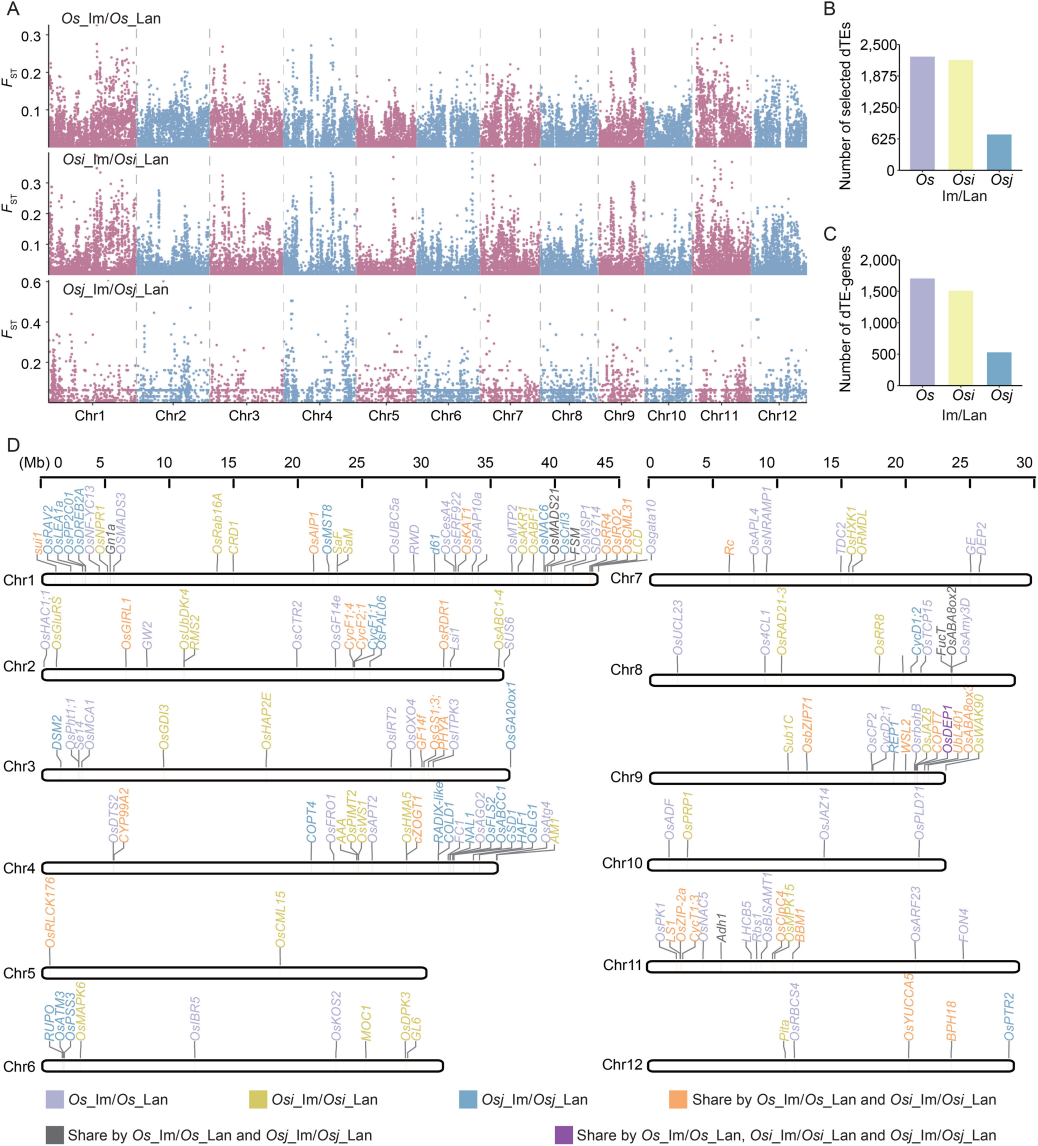
Contribution of TE variation to divergent selection during rice improvement. **(A)** Selection signatures in comparisons between landraces and improved varieties of the *Os*, *Osi*, and *Osj* accessions based on TE variations. **(B)** Number of dTEs exhibiting signatures of selection (i.e., ranked in the top 5% of *F*_ST_ value, termed “selected dTEs”) between landraces and improved varieties in the *Os*, *Osi*, and *Osj* accessions. *F*_ST_ outliers based on dTEs are indicated as loci showing signatures of selection. **(C)** Number of genes harboring selected dTEs within their genic regions (termed “dTE-genes”) between landraces and improved varieties in the *Os*, *Osi*, and *Osj* accessions. Genic regions included the gene body, 2 kb upstream (promoters) and 2 kb downstream (downstream) regions. **(D)** Positions of functional dTE-genes on chromosomes 1-12.

To identify selective signals associated with dTEs during rice improvement, we estimated population divergence between landrace and improved varieties using *F*_ST_ analysis (**Figure 2A**). By ranking the top 5% of the *F*_ST_ values, we detected a total of 4,334 selected dTEs, including 2,258, 2,192, and 711 selective dTEs in pairwise comparisons of *Os*_im vs. *Os*_lan, *Osi*_im vs. *Osi*_lan, and *Osj*_im vs. *Osj*_lan, respectively (**Figure 2B**), suggesting their potential involvement in the genetic improvement from landraces to improved varieties.

We analyzed genes overlapping with selective dTEs (within ± 2 kb of a gene body) and identified 3,070 potential candidate genes (henceforth termed “dTE-genes,” **Figure 2C**) whose functions or expressions could be affected by dTEs. These included 1,703 dTE-genes with evidence of selection in *Os*_im vs. *Os*_lan, 1,503 in *Osi*_im vs. *Osi*_lan, and 530 in *Osj*_im vs. *Osj*_lan. Gene Ontology (GO) enrichment analysis revealed that the dTE-genes were significantly enriched in biological processes related to basic metabolism and development and the response to abiotic and biotic stress (*p <* 0.05, **Supplementary Figure S4**). Among them, 154 genes were previously reported to be associated with plant growth, panicle and grain development, and resistance to heat, chilling, and salt stress (**Figure 2D**). Notable examples include *GW2*, which regulates grain width and weight; *Gn1a*, associated with grain number; *OsMCA1*, which regulates plant architecture; and *COLD1*, which regulates chilling tolerance in japonica rice.

### TE variations associated with gene expression change during rice improvement

**Figure 3:**
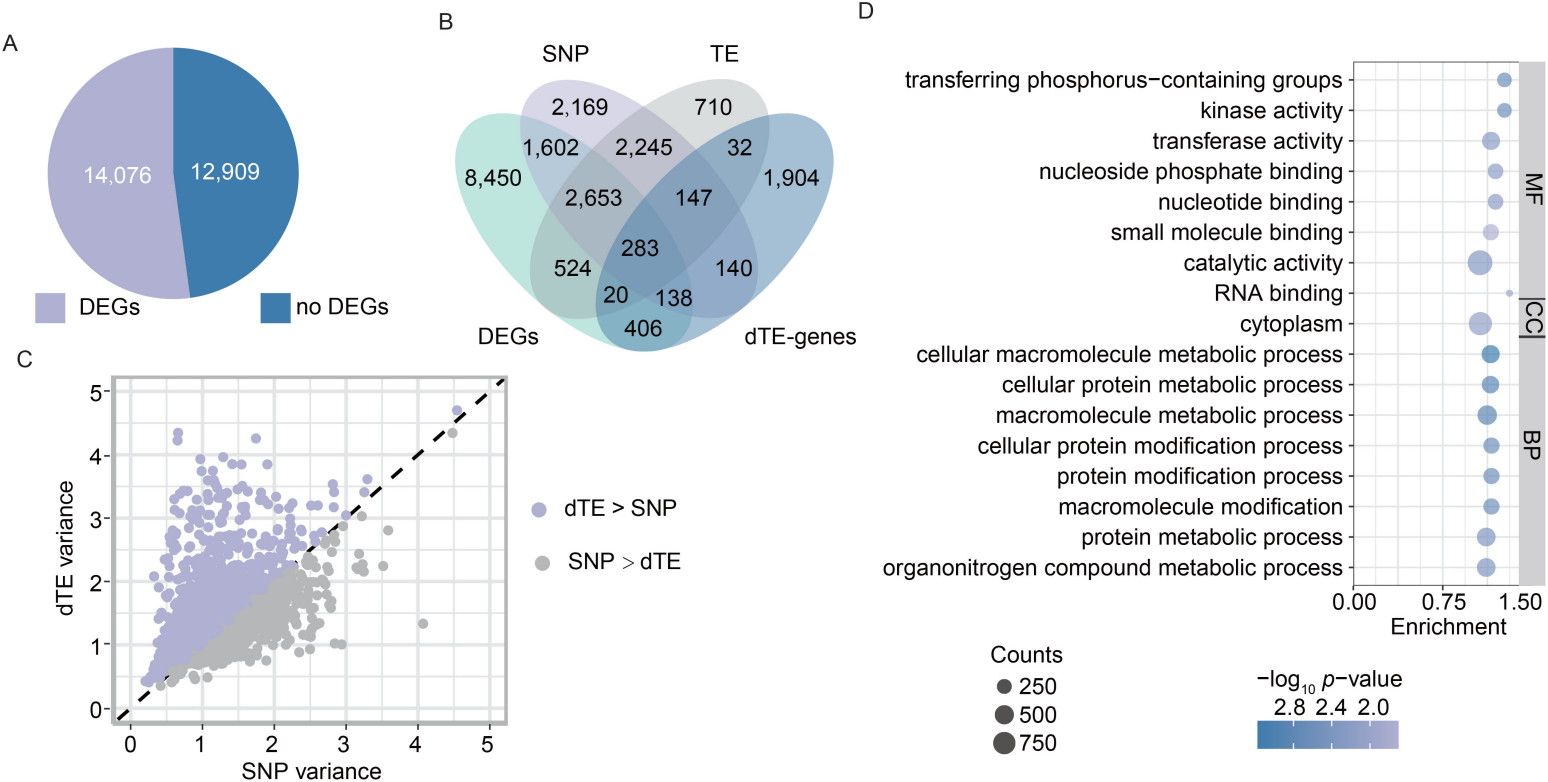
TE variations associated with gene expressions during rice improvement. **(A)** Number of differentially expressed genes (DEGs) across the genome. DEGs between landraces and improved varieties were identified using a threshold of *p-*value < 0.05. **(B)** Statistical comparison of dTE-genes, DEGs, and eGenes associated with dTEs and SNPs. eGenes are genes whose expression is significantly associated with dTEs and SNPs between landraces and improved varieties. **(C)** Expression variance explained by leading dTE-eQTL and SNP-eQTL. Each point represents a gene. **(D)** GO analysis of the 3,480 eGenes (i.e., DEGs) associated with dTEs between landraces and improved varieties. BP, CC, and MF refer to biological process, cellular component, and molecular function, respectively.

To further elucidate the role of TEs in rice improvement, we conducted comparative analysis of transcriptional dynamics across landraces and improved varieties, with subsequent evaluation of the regulatory contribution derived from the dTEs to observed expression divergence. Transcriptome data of young leaves and panicles for the 192 *Os* accessions were obtained from previous studies (Shang et al., 2022; Zhang et al., 2024). Differentially expressed genes (DEGs) between landraces and improved varieties were identified using a threshold of *p* < 0.05. We detected a total of 14,076 DEGs across the whole genome (**Figure 3A**). Among them, 3,917 and 5,455 DEGs exhibited upregulation in young leaves and panicles of improved varieties relative to landraces, whereas 5,735 and 4,919 DEGs demonstrated downregulation in these corresponding tissues (**Supplementary Figure S5A**). Functional enrichment analysis indicated that upregulated DEGs in leaves were primarily involved in the regulation of metabolic processes (**Supplementary Figure S5B**), whereas downregulated DEGs were enriched in biosynthesis, basic metabolic processes, signal transduction, and cellular responses to stimuli (**Supplementary Figure S5C**). In young panicles, upregulated DEGs were associated with gene expression, biosynthesis, and basic metabolic processes (**Supplementary Figure S5D**), while downregulated DEGs were linked to primary metabolic and developmental processes, signal transduction, and cellular responses to stimuli (**Supplementary Figure S5E**). These DEGs likely drive phenotypic changes during rice improvement, primarily through various variations, such as TE, SNP, and other variations.

TEs can be inserted or deleted near or within genes, altering gene expression and rewiring gene regulatory networks through epigenetic mechanisms and transcription factor-binding sites. To evaluate the relative contributions of dTEs and SNPs to gene expression changes, we associated dTEs and SNPs with gene expression levels among *Os* accessions and identified the dTE- and SNP-based expression quantitative trait loci (eQTLs), respectively. Using transcriptome data from young panicles, we identified 3,572 genes whose expression was significantly associated with dTEs (referred to as dTE-based eGenes). Combining these with 3,868 eGenes previously identified from leaf transcriptomes (Li et al., 2024), we obtained a total of 6,614 dTE-based eGenes. Among them, 3,480 were overlapped with DEGs between landraces and improved varieties, accounting for 24.7% of the DEGs (**Figure 3B**), including 2,512 (24.9%), 1,534 (22.8%), and 1,075 (23.3%) of the DEGs in *Os*_lan vs. *Os*_im, *Osi*_lan vs. *Osi*_im, and *Osj*_lan vs. *Osj*_im, respectively. We further compared the contributions of dTEs and SNPs to gene expression levels in the *Os* accessions by analyzing dTE- and SNP-based eQTLs. Among the 2,936 eGenes shared between TE-eQTL and SNP-eQTL analyses (**Figure 3B**), leading dTEs explained more expression variance than leading SNPs for approximately 60.9% of these genes (**Figure 3C**). Notably, 544 out of 3,480 DEGs exhibited expression variation associated exclusively with dTEs (**Figure 3B**), underscoring the importance of considering TE variations in rice gene expression studies. Functional enrichment analysis revealed that these genes were significantly enriched in biological processes related to basic metabolic processes (**Figure 3D**). These results imply the potentially significant contribution of TE variations to the changes in gene expression patterns between landrace and improved varieties in rice improvement.

### Candidate genes influenced by TE variations for genetic improvement in rice

By integrating selective dTE-genes, DEGs, and dTE-based eGenes, we identified 303 genes that are shared across all three datasets (**Figure 3B**), which represented important candidates potentially involved in rice improvement processes. For example, we observed that the improved varieties of *Osi* exhibited significantly narrower grain widths compared to landraces, which may represent a critical enhancement in appearance quality traits for indica rice (**Supplementary Figure S6A**). The expression of *LOC_Os03g11790*, a DEG associated with seed size regulation and secondary metabolism (Zhang et al., 2019a), was significantly upregulated in the *Osi_*im accessions relative to *Osi*_lan accessions (**Supplementary Figure S6B**). This gene is a dTE-based eGene, whose expression is significantly influenced by a selective dTE, specifically a 178-bp TE deletion (DEL) located downstream region of this gene (**Supplementary Figures S6C, D**). *Osi* accessions harboring the derived DEL genotype exhibited a significantly narrower grain width compared to those with the ancestral genotype (**Supplementary Figure S6E**), indicating that positive selection of this dTE may have contributed to the reduction in rice grain width from landrace to improved varieties in indica rice. Additionally, this dTE was absent in the *Osj* accessions **Supplementary Figure S6C**), indicating its limited impact on agronomic traits in japonica rice.

Furthermore, we also observed significantly shorter plant heights in the *Osi*_im accessions compared to the *Osi*_lan accessions (**Supplementary Figure S7A**), likely a result of dwarf breeding during the First Green Revolution. The expression of *LOC_Os09g11520*, a DEG implicated in rice growth and development (Luo et al., 2024), was significantly upregulated in the *Osi*_im accessions compared to the *Osi*_lan accessions (**Supplementary Figure S7B**). A selective dTE, specifically a 430-bp DEL of *Helitron*, was identified as a local eQTL for this gene and was located in its downstream region (~1.9 kb) (**Supplementary Figure S7C**). Rice accessions with this dTE exhibited significantly higher expression levels of *LOC_Os09g11520* (**Supplementary Figure S7D**) and shorter plant heights (**Supplementary Figure S7E**) across the *Os*_lan, *Os*_im, *Osi*_lan, and *Osi*_im accessions, indicating the potential contribution of this positively selected dTE to the reduction in rice plant height.

**Figure 4:**
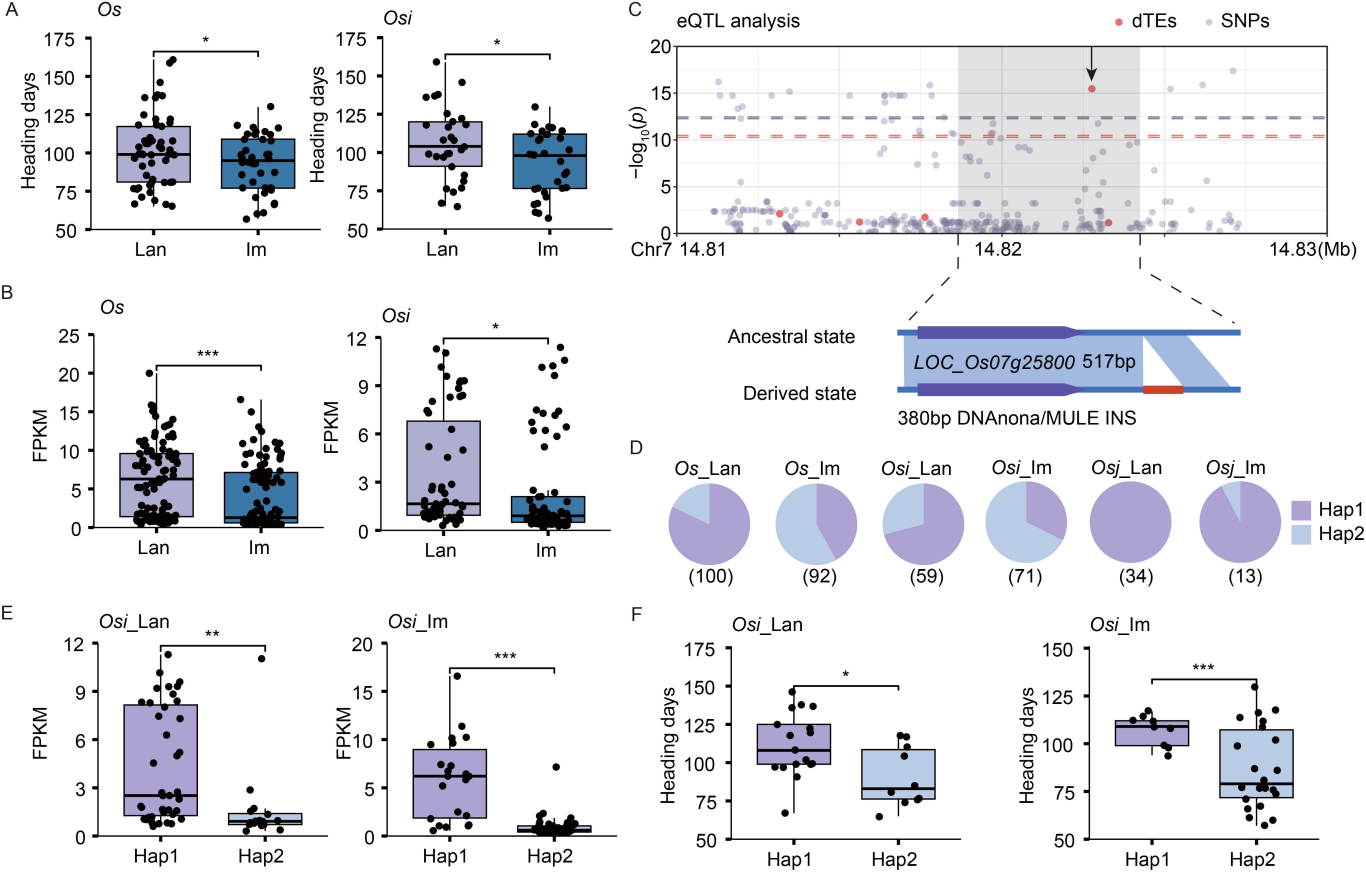
dTE associated with heading days in landraces and improved varieties. **(A, B)** Differences in heading days **(A)** and expression levels of *LOC_Os07g25800* **(B)** between landraces and improved varieties. **(C)** Manhattan plot of *LOC_Os07g25800* expression levels, dTE variants, and SNPs (top). The leading dTE (380 bp insertion, INS) associated with *LOC_Os07g25800* expression is indicated (bottom). **(D)** Distribution of the dTE across different subpopulations. The total number of accessions analyzed is listed below the pie chart. **(E, F)** Differences in the expression levels of *LOC_Os07g25800* **(E)** and heading days **(F)** between the accessions with (derived state, Hap2) and without (ancestral state, Hap1) the dTE INS event in the *Osi*_lan and *Osi*_im accessions. Significance was determined by the Student’s *t*-test, ***p < 0.001, **p < 0.01, *p < 0.05.

After excluding SNP-based eGenes, we further identified 20 candidate genes that were exclusively influenced by dTEs. For example, *Os*_im and *Osi*_im accessions exhibited fewer days to heading compared to the *Os*_lan and *Osi*_lan accessions (**Figure 4A**). The expression of *LOC_Os07g25800*, a DEG known to mediate responses to environmental stimuli (Luo et al., 2024; Cao et al., 2008), showed significantly lower expression levels in the *Os*_im and *Osi*_im accessions compared to the *Os*_lan and *Osi*_lan accessions, respectively (**Figure 4B**). Expression variation of *LOC_Os07g25800* was significantly associated with a selective dTE INS event in its downstream region, with no associated SNPs detected (**Figure 4C**). This dTE INS was present in nearly half of the improved varieties (50% and 64.8% in *Os*_im and *Osi*_im accessions, respectively), only a few landraces (16% and 27.1% in *Os*_lan and *Osi*_lan accessions, respectively), and was absent in the *Osj* and *Or* accessions (**Figure 4D**, **Supplementary Figure S8A**). Accessions with this dTE INS exhibited significantly lower expression levels of *LOC_Os07g25800* (**Figure 4E**, **Supplementary Figure S8B**) and shorter heading days compared with those without it in the *Osi*_lan, *Os*_im, *Osi*_lan, and *Osi*_im accessions (**Figure 4F**, **Supplementary Figure S8C**).

**Figure 5:**
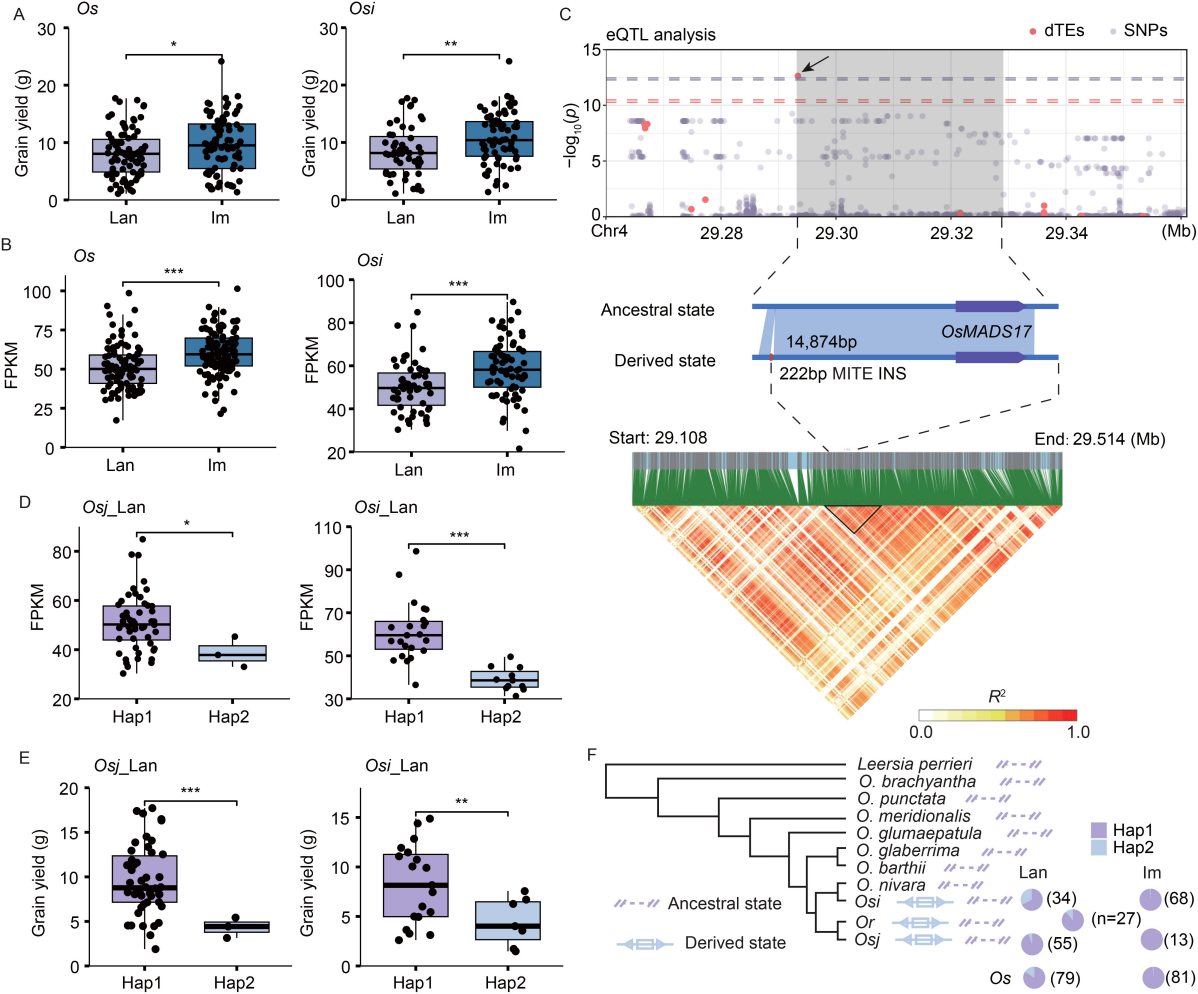
TE variations associated with grain yield per plant in landraces and improved varieties. **(A, B)** Differences in grain yield per plant **(A)** and expression levels of *OsMADS17* (*LOC_Os04g49150*) **(B)** between landraces and improved varieties. **(C)** Manhattan plot of *OsMADS17* expression levels, dTE variants, and SNPs (top). The leading dTE (222 bp insertion, INS) associated with *OsMADS17* expression is indicated (middle). Linkage disequilibrium heatmap of the genomic region containing *OsMADS17* and its upstream TE variation (bottom). **(D, E)** Differences in expression levels of *OsMADS17* **(D)** and grain yield per plant **(E)** between the accessions with (derived state, Hap2) and without (ancestral state, Hap1) the dTE INS event in the *Osi*_lan and *Osj*_lan accessions. Significance was determined using Student’s *t*-test, ***p < 0.001, **p < 0.01, *p < 0.05. **(F)** A dTE INS in the upstream region of *OsMADS17* was detected in several *Osi*_lan (11 accessions), *Osi*_im (one accession), *Osj*_lan (three accessions), and *O. rufipogon* (*Or*, three accessions) accessions. However, it was absent in *Osj*_im accessions and the rice outgroup genomes CC and EE.

Grain yield per plant is a critical trait in rice improvement, and the *Os*_im and *Osi*_im accessions demonstrated significantly higher grain yields compared to the *Os*_lan and *Osi*_lan accessions (**Figure 5A**). The expression levels of *OsMADS17* (*LOC_Os04g49150*), a DEG associated with grain yield by regulating floral organ identity and meristem fate (Hu et al., 2015), were significantly upregulated in the *Os*_im and *Osi*_im accessions compared to the *Os*_lan and *Osi*_lan accessions (**Figure 5B**). A 222-bp dTE (*Tourist* MITE) INS located upstream of *OsMADS17* (**Figure 5C**) was associated with expression variation of this gene (**Figure 5D**). The accessions without this dTE (the ancestral genotype) showed significantly higher grain yields in both the *Osi*_lan and the *Osj*_lan accessions than those with it (**Figure 5E**), suggesting the deleterious effect of this dTE to the grain yield. This deleterious dTE showed a substantial decrease in the population frequency from *Os*_lan (15.7%) to *Os*_im (1.2%) (**Figure 5F**), indicating a potential advantage of the ancestral allele from wild rice in the rice improvement.

## Discussion

Rice is a major food crop for billions of people worldwide (Wing et al., 2018) and serves as a model species for monocots and crop plants. The domestication of rice commenced approximately 10,000 years ago, with a focus on domestication-related traits such as reduced seed shattering, increased grain size, modified plant architecture, altered awn length, and enhanced seed dormancy (Huang and Han, 2015). In contrast, the improvement has been ongoing since the mid-twentieth century, aimed at improving critical agronomic traits, including plant height, yield potential, stress tolerance, growth duration, and grain quality (Stone and Glover, 2017). An in-depth understanding of rice phenotypic variation during the domestication and improvement processes through multi-omics technologies will significantly benefit agriculture, world food security, and biological and genomic research communities. Despite the widespread utility of SNPs in rice genetics research, structural variations such as TE variations have been largely overlooked. As pan-genomes become widely available for rice (He et al., 2025), TEs, as a key driver of genomic structural variation, receives greater attention in elucidating the genetic mechanisms underlying rice domestication. Previous studies have utilized Illumina short-read sequencing data to investigate TE variations in cultivated rice accessions, aiming to trace the rice domestication history (Carpentier et al., 2019) and to explore the associations between TEs and both agronomic traits (Yan et al., 2022) and gene expressions (Castanera et al., 2023). Li et al. examined the TE variations across wild and cultivated rice accessions using high-quality genome assemblies to evaluate their contribution to rice domestication (from *Or* to *Osi* and from *Or* to *Osj*) and differentiation (between *Osi* and *Osj*),and identified candidate genes that may play crucial roles in this key process of rice speciation (Li et al., 2024).

In this study, we further investigated the contribution of TE variations to rice improvement from landraces to modern improved varieties, focusing on the secondary revolutionary advancements in important agronomic traits such as plant height, grain width, grain yield, and heading date. By integrating a high-quality pan-TE map and population-scale transcriptome data from young leaves and panicles, our analysis revealed significant differences in TE variants abundance and their associated gene expression patterns between landraces and improved varieties. For example, improved varieties exhibited higher numbers of Ty3-retrotransposons, LTR *Copia*, *Helitron*, and DTC family variations compared to landraces, while SINE family variations were less abundant (**Supplementary Figure S3D**) This discrepancy likely contributes to phenotypic diversity, although the small size and high copy number of SINE elements pose challenges for accurate analysis (Domínguez et al., 2020).

We identified 3,070 potential dTE-genes that may regulate phenotypic variation between landraces and improved varieties, with 2,448 of these undetectable during domestication from *Or* to *Os* accessions (Li et al., 2024). Given that TE variations often explain more phenotypic variance than SNPs and can enhance genomic prediction accuracy (Vourlaki et al., 2022), we conducted a dTE-based eQTL analysis. We found that dTEs explained 60.9% more expression variance than SNPs, surpassing the effect of TE insertions (Castanera et al., 2023). This analysis detected 544 dTE-based eGenes from DEGs, with their expression variation exclusively linked to TE variations (absent in SNP-based eGenes), highlighting the unique role of TEs in gene regulation. Notably, we observed a substantial increase in frequency of the beneficial dTEs in the improved varieties compared with landraces (**Figure 4D**, **Supplementary Figure S6C**, **S7C**), enhancing the potential contribution of those selective dTEs to rice improvements. Some deleterious TE variants, such as a 222-bp dTE (*Tourist* MITE) insertion on upstream of *OsMADS17*, were restored to their ancestral status in the improved varieties (**Figure 5F**), underscoring the potential of wild rice-derived alleles in enhancing agronomic traits.

Among the 14,076 DEGs identified between landraces and improved varieties, approximately 24.7% and 33.2% were regulated by SNPs and TE variations, respectively. The remaining DEGs may be also influenced by other genetic variations, such as inversions (He et al., 2024a), tandem repeats (He et al., 2024b), and rare variants (Wang et al., 2023). The limited number of *Osj*_im accessions (n=16) may constrain the exploration of TE variations and subsequent population analyses. Expanding the *Osj*_im population and generating reference-quality genomes will address this limitation (Shang et al., 2023).

The dTE markers identified in this study represent valuable genetic resources for gene mining, genome editing, and breeding programs (Zhang et al., 2019b). By leveraging dTE markers, breeders can assess the genetic potential of landrace and improved rice parents to develop superior hybrids. These findings underscore the significance of TEs in rice improvement and provide a foundation for future genetic and breeding research.

## Materials and methods

### Materials and data collection

A high-quality pan-TE map for 221 Asian rice accessions (including 100 *Os*_lan accessions and 92 *Os*_im accessions and 29 *Or* accessions) was obtained from our previous study (Li et al., 2024) and downloaded from NCBI under PRJNA656318. TE variations were defined as derived TE variations (dTEs) exhibiting both ancestral and derived states among rice accessions relative to the outgroups (including one *O. glaberrima*, one *O. barthii*, and one *O. glumaepatula*). The ancestral state indicates that the genotype of the TE variation in a given accession (0/0) is the same as that in outgroups (0/0), whereas the derived state indicates that the genotype of the TE variation in a given accession (1/1 or 0/1) is different from that in outgroups (0/0). Transcriptome data for 192 *Os* leaves and 192 young panicles were obtained from previous studies (Shang et al., 2022; Zhang et al., 2024) and downloaded from NCBI under PRJNA692672 and PRJNA682327, respectively. Detailed information on these accessions is provided in **Supplementary Table S1**.

The variable importance ranking for the phenotypic data between rice landraces and improved varieties was conducted by employing a random forest model in the R environment. The MICE (van Buuren and Groothuis-Oudshoorn, 2011) package was used to conduct the imputation of the missing values in the phenotypic data via multiple imputation methods. The dataset was stratified into training and testing subsets (8:2 ratio) for model development. The hyperparameter tuning was performed through a grid search with a nested fivefold cross-validation, selecting optimal parameters (mtry = 2, ntree = 1,000, min.node.size = 1) that minimized the out-of-bag (OOB) error. The random forest model was trained with the optimal hyperparameters. Model performance was evaluated through confusion matrices (such as predicting accuracy, sensitivity, and specificity), AUC statistics, and variable importance rankings. These analyses were implemented using packages ranger (Wright and Ziegler, 2017), randomForest (Liaw and Wiener, 2002), and caret (Kuhn, 2008), with class imbalance addressed through stratified sampling and balanced subsampling strategies.

### Selective sweeps

The relative divergence measure (*F*_ST_) was employed to identify the divergent regions between the landraces and improved varieties, including comparisons of *Os*_lan vs. *Os*_im, *Osi*_lan vs. *Osi*_im, and *Osj*_lan vs. *Osj*_im. *F*_ST_ values were calculated for each TE variation using VCFtools (version 0.1.16, Danecek et al., 2011), and TE variations ranking in the top 5% of *F*_ST_ values were designated as highly divergent regions (Shang et al., 2022).

### eQTL analysis

Gene expression data for leaves and TE-based expression quantitative trait loci were obtained from our previous study (Li et al., 2024). For young panicles, TE variants of 192 *Os* accessions were filtered out using VCFtools (Danecek et al., 2011) with the parameters “–maf 0.05 –max-missing 0.2.” Population structure was inferred through principal component analysis (PCA) using Plink2 (version 2.00a3LM, parameters: -pca 10) (Chen et al., 2019). The first 10 principal components from PCA and 20 factors from the probabilistic estimation of expression residuals (PEER) were included as covariates. Associations between TE-gene pairs in young panicles were detected using the linear regression model implemented in the MatrixEQTL package (version 2.2) (Shabalin, 2012). *p*-Values were corrected by the Benjamini-Hochberg method at α = 0.05, with a genome-wide error threshold of *p* = 3.252e−11 (calcuated using the R package P. adjust, version 3.1.2, Benjamini and Yekutieli, 2001).

### Functional enrichment analysis

GO enrichment analysis was performed to assess biological significance, including biological processes (BP), cellular components (CC), and molecular functions (MF), using TBtools (version 1.108) (Chen et al., 2020). Background and annotation information were obtained from the eggNOG (http://eggnogdb.embl.de/#/app/emapper) and GENEONTOLOGY (http://geneontology.org/docs/download-ontology/) databases. Significance was defined as *p* < 0.05, and GO visualization was achieved using the R package ggplot2.

### Linkage disequilibrium analysis

Linkage disequilibrium (LD) was measured for a genomic region (29.10-29.51 Mb on chromosome 4) containing a 222-bp TE (*Tourist* MITE) INS near the MADS-box family gene *OsMADS17*. SNPs, InDels, and TE variations were filtered using VCFtools (Danecek et al., 2011) with parameters –maf 0.05 –max-missing 0.2. For each TE variation, the maximum *R^2^* value with adjacent SNPs and InDels within 50 kb on either side was calculated using Plink2 (parameters: –r2 –ld-window-r2 0 –ld-window-kb 50000) (Chen et al., 2019).

### Phenotype collection

We collected the phenotypic data for heading days, plant height and grain width of all *Os* accessions from our previous studies (He et al., 2024b; Li et al., 2024). Grain yield per plant was measured at maturity for each accession, with data collected from six plants per accession. Field experiments were conducted in Mianyang, Sichuan Province, China, in 2020.

### Statistical analysis

Student’s *t*-tests (two-tailed) were used to compare data between groups, and a hypergeometric test (phyper function in R) was performed to assess the enrichment.

## Data Availability

The original contributions presented in the study are included in the article/**Supplementary Material**. Further inquiries can be directed to the corresponding authors.
