# Supplementary material for: Uncovering the breeding contribution of transposable elements from landraces to improved varieties through pan-genome-wide analysis in rice
Source: Front Plant Sci. 2025 Apr 14;16:1573546. doi: 10.3389/fpls.2025.1573546 (PMC12034714; doi:10.3389/fpls.2025.1573546)
Supplement: Supplementary file 1 [file DataSheet1.pdf]

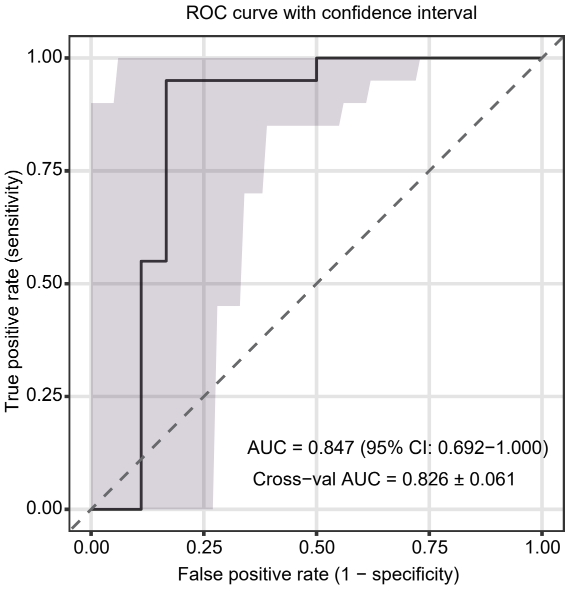

Fig. S1 Receiver Operating Characteristic (ROC) curve for the random forest classifier. ROC curve demonstrating the classification performance of the optimized random forest model in distinguishing rice landraces from improved varieties. The diagonal dashed line represents random classification performance. Shaded regions indicate 95% confidence intervals of the ROC curve. The area under the curve (AUC) value, along with its standard deviation from 5-fold cross-validation, is displayed in the lower right.

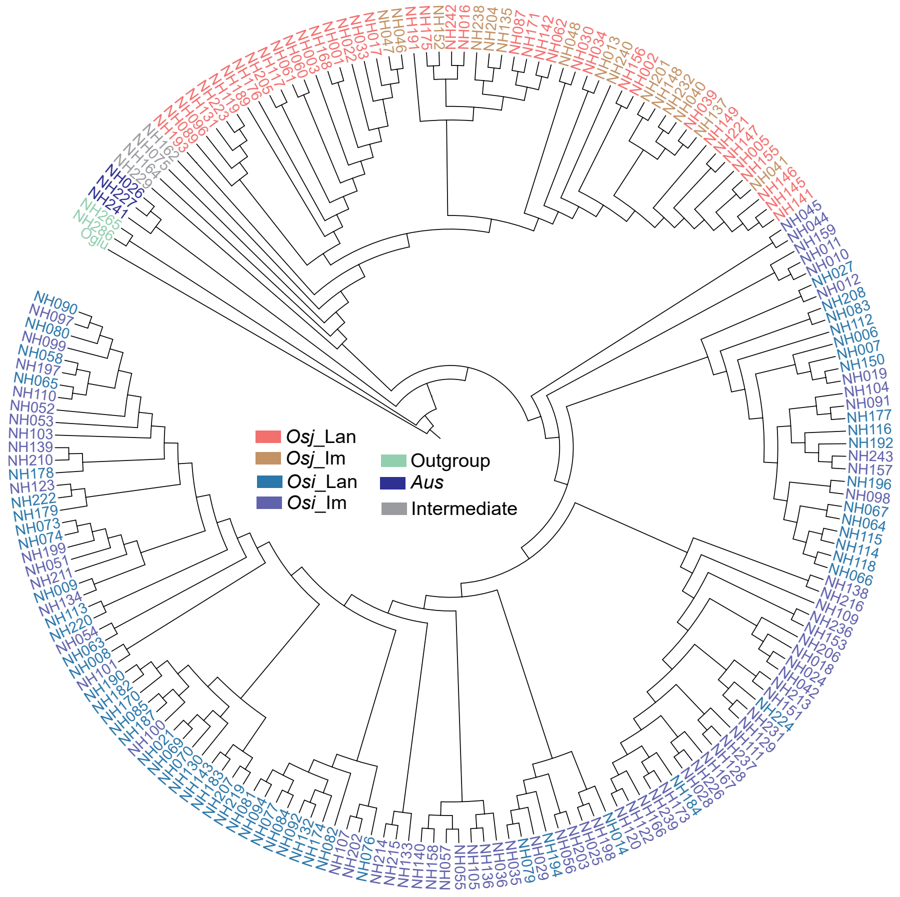

Fig. S2 Phylogeny of 192 *Os* and three outgroup accessions based on whole-genome SNPs. *Osi*_lan, *Osi*_im, *Osj*_lan, and *Osj*_im refer to *Osi* landraces, improved varieties of *Osi*, *Osj* landraces, and improved varieties of *Osj*, respectively. *Os*, *Osi*, *Aus*, *Osj*, and outgroup respectively refer to *O. sativa*, *O. sativa indica*, *O. sativa aus*, and *O. sativa japonica*, respectively. Outgroup included one *O. glaberrima*, one *O. barthii*, and one *O. glumaepatula*.

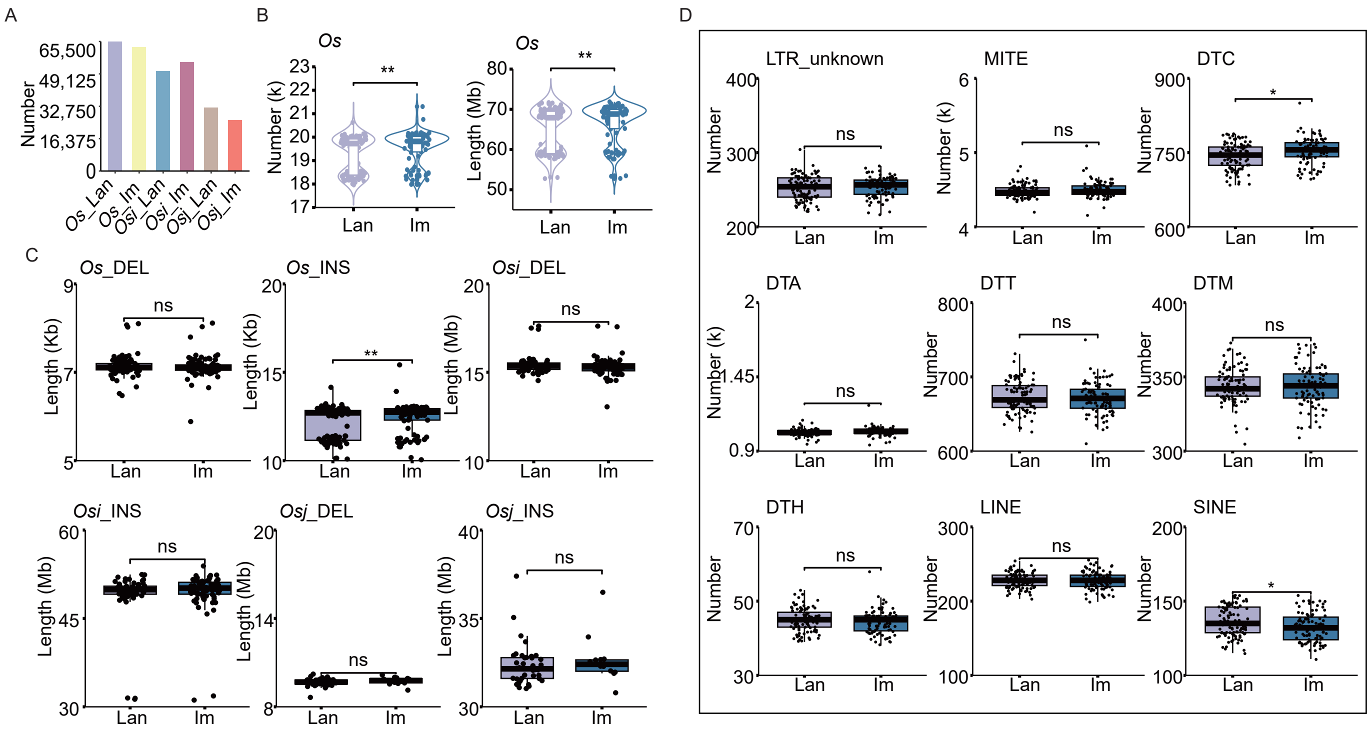

Fig. S3 Differences in TE variation between rice landraces and improved varieties. A Total number of non-redundant dTEs and sequences detected across different subpopulations. *Os*, *Osi*, and *Osj* refer to *O. sativa*, *O. sativa indica*, and *O. sativa japonica*, respectively. *Os*_lan, *Os*_im, *Osi*_lan, *Osi*_im, *Osj*_lan, and *Osj*_im refer to *Os* landraces, improved varieties of *Os*, *Osi* landraces, improved varieties of *Osi*, *Osj* landraces, and improved varieties *Osj*, respectively. B Differences in the total number and length of TE variations per accession across different rice subpopulations. C Differences in the number of TE insertion (INS) and deletion (DEL) in each landrace and improved variety across different subpopulations. DEL and INS refer to deletion and insertion events identified by comparing the Asian accessions to the outgroups (*O. glaberrima*, *O. barthii*, and *O. glumaepatula*), respectively. D Differences in the number of dTEs across TEs families in each landrace and improved variety. Significance was determined using Student's *t*-test, $^{**}P < 0.01$, $^{*}P < 0.05$.

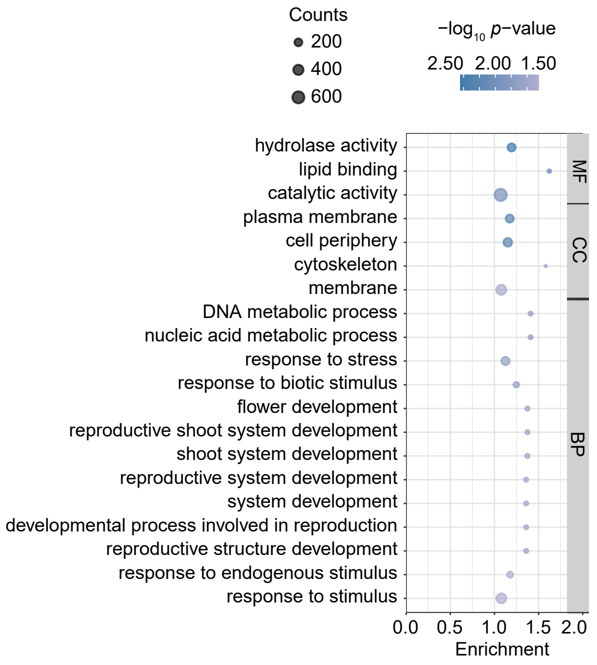

Fig. S4 Gene Ontology (GO) analyses of dTE-genes. BP, CC, and MF refer to biological process, cellular component, and molecular function, respectively.

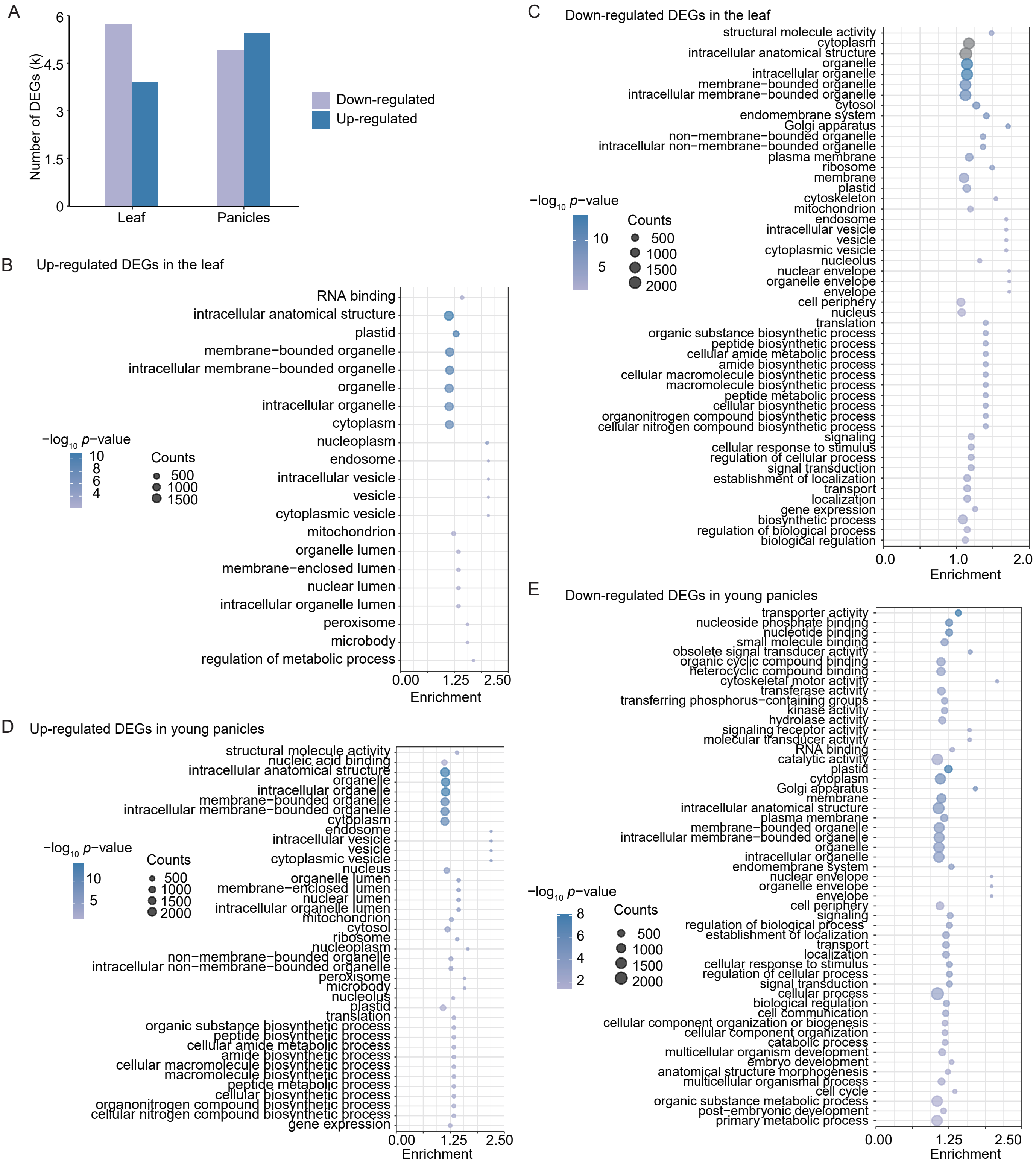

Fig. S5 Differentially expressed genes between rice landraces and improved varieties. A Number of differentially expressed genes (DEGs) between landraces and improved varieties in leaves and young panicles. DEGs were identified using a threshold of $P < 0.05$. Up-regulated and down-regulated DEGs in improved varieties are relative to those in landraces. B-C Gene Ontology (GO) analysis of up-regulated (B) and down-regulated DEGs (C) in improved varieties relative to landraces in leaves. BP, CC, and MF refer to biological process, cellular component, and molecular function, respectively. D-E GO analysis of up-regulated (D) and down-regulated DEGs (E) in young panicles of improved varieties relative to landraces .

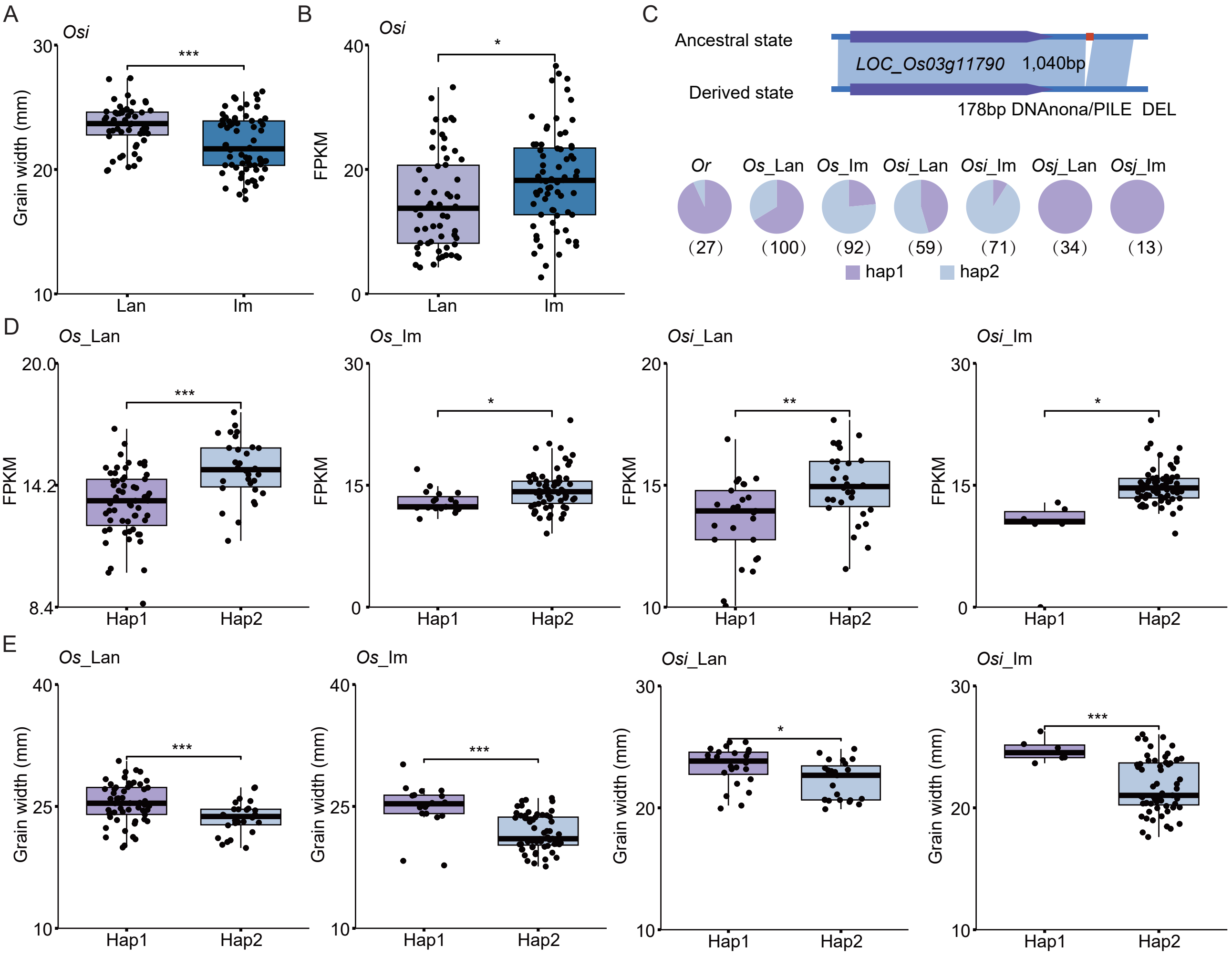

Fig. S6 dTE associated with grain width in rice landraces and improved varieties. A-B Differences in grain width (A) and expression levels of *LOC_Os03g11790* (B) between landraces and improved varieties in *Osi* accessions. C A dTE deletion (DEL) located downstream of *LOC_Os03g11790* (top). Distribution of the dTEs across different subpopulations. The total number of accessions analyzed is listed below the pie chart. D-E Differences in expression levels of *LOC_Os03g11790* (D) and grain width (E) between accessions with (derived state, Hap2) and without (ancestral state, Hap1) the dTE DEL events. Significance was determined using Student's *t*-test, ****P* < 0.001, ***P* < 0.01, **P* < 0.05.

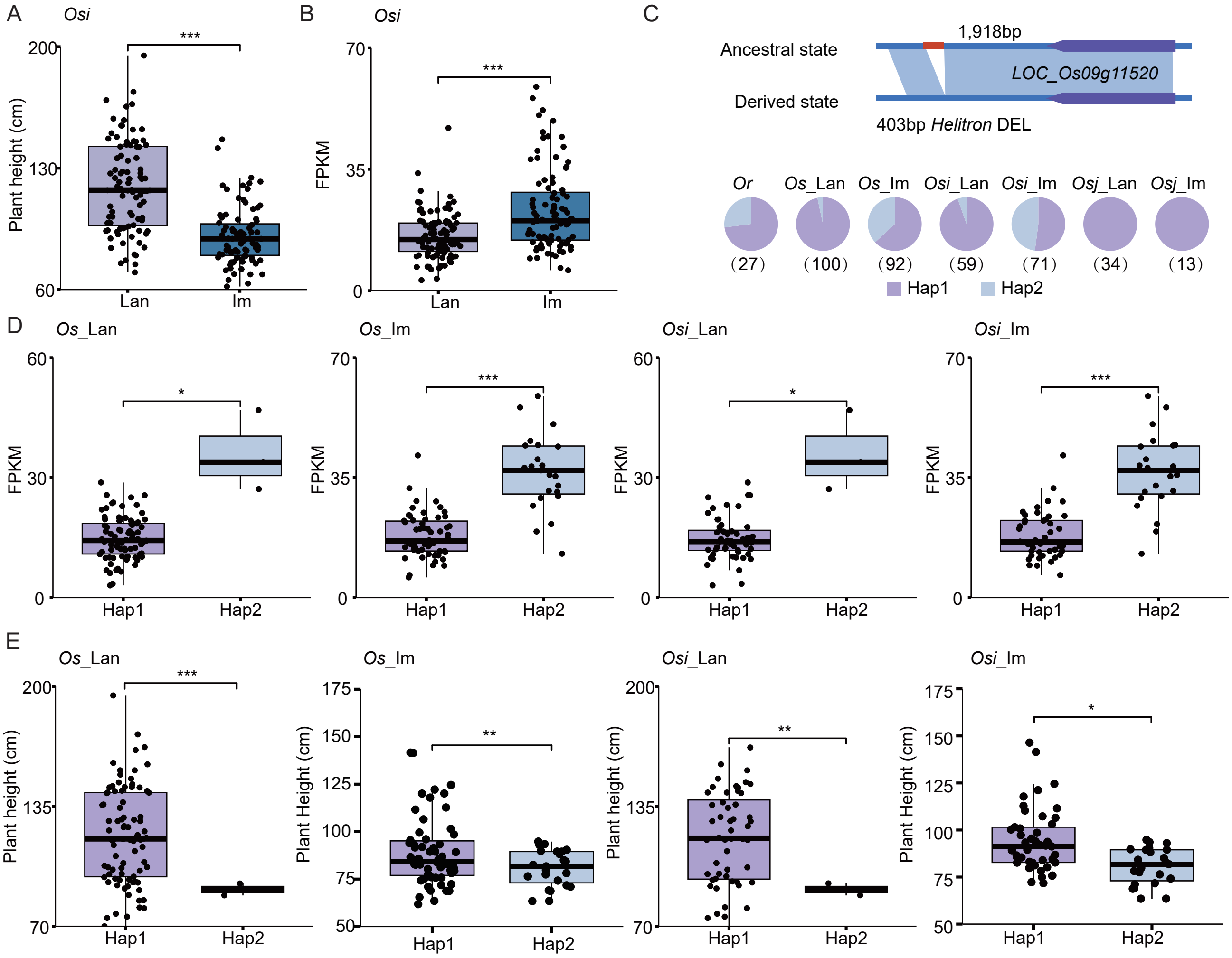

Fig. S7 dTE associated with plant height in rice landraces and improved varieties. A-B Differences in plant height (A) and expression levels of *LOC_Os09g11520* (B) between landraces and improved varieties in *Osi* accessions. C A helitron deletion (DEL) occurred in the downstream of *LOC_Os09g11520*. Distribution of the dTE in different subpopulations. The total number of accessions analyzed is listed below the pie chart. D-E Differences in expression levels of *LOC_Os09g11520* (D) and plant height (E) between the accessions with (derived state, Hap2) and without (ancestral state, Hap1) the dTE DEL events. Significance was determined using Student's *t*-test, ****P* < 0.001, ***P* < 0.01, **P* < 0.05.

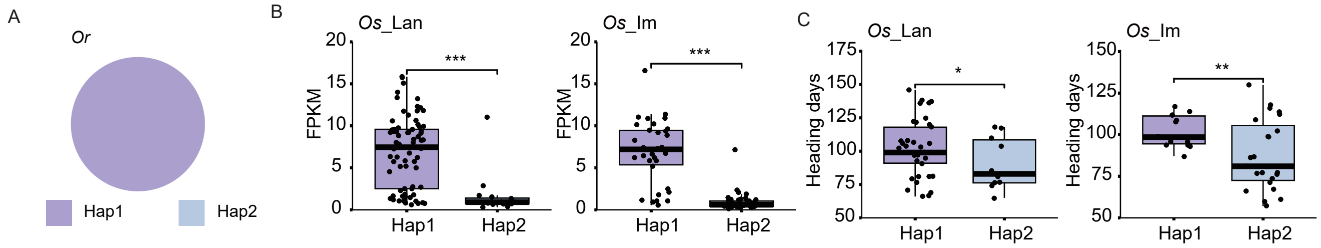

Fig. S8 dTE associated with heading days in rice landraces and improved varieties. A Distribution of the TE INS downstream of *LOC_Os07g25800* in *Or* accessions (n=27). B-C Differences in expression levels of *LOC_Os07g25800* (B) and heading days (C) between the accessions with (derived state, Hap2) and without (ancestral state, Hap1) the dTE INS events . Significance was determined using Student's *t*-test, ***$P < 0.001$, **$P < 0.01$, *$P < 0.05$.
